# The causal association between gut microbiota and postpartum depression: a two-sample Mendelian randomization study

**DOI:** 10.3389/fmicb.2024.1415237

**Published:** 2024-09-02

**Authors:** Wenjun Jin, Bo Li, Lijun Wang, Lin Zhu, Songhao Chai, Rui Hou

**Affiliations:** ^1^Medical Department, Sias University, Zhengzhou, Henan, China; ^2^Medical Department, Zhengzhou University of Industry Technology, Zhengzhou, Henan, China; ^3^Ultrasound Department, The First Affiliated Hospital of Zhengzhou University, Zhengzhou, China

**Keywords:** gut microbiota, postpartum depression, Mendelian randomization, instrumental variables (IVs), single nucleotide polymorphisms (SNPs)

## Abstract

**Background:**

An escalating body of clinical trials and observational studies hints at a plausible link between gut flora and postpartum depression (PPD). The definitive causal dynamics between these two entities remain shrouded in ambiguity. Therefore, in this study, we employed the two-sample Mendelian randomization approach to ascertain the causal link between gut microbiota and PPD.

**Methods:**

Summary-level GWAS data related to the human gut microbiota were obtained from the international consortium MiBioGen and the Dutch Microbiome Project (species). For PPD, GWAS data were derived from the FinnGen biobank, consisting 57,604 cases and 596,601 controls. The inverse variance weighted method (IVW) as the cornerstone of our analytical approach. Subsequent to this, a comprehensive suite of tests for pleiotropy and heterogeneity were conducted to ensure the reliability and robustness of our findings.

**Results:**

We identified 12 bacterial taxa associated with the risk of PPD. Veillonellaceae, *Ruminococcaceae UCG 011*, *Bifidobacterium adolescentis*, *Paraprevotella clara*, *Clostridium leptum*, *Eubacterium siraeum*, *Coprococcus catus* exhibited an inversely associated with the risk of PPD. Alphaproteobacteria, *Roseburia*, *FamilyXIIIAD3011group*, *Alistipes onderdonkii*, *Bilophila wadsworthia* showed a positive correlation with the risk of PPD.

**Limitations:**

The GWAS data derived from the MiBioGen consortium, DMP, and FinnGen consortium, may introduce selection bias. Moreover, the data primarily originates from European populations, hence extrapolating these results to diverse populations should be approached with caution. The etiological factors behind PPD remain enigmatic, alluding to the existence of potential undisclosed confounders.

**Conclusion:**

Based on this MR analysis, we found a causal relationship between certain gut microbial communities and PPD. Future clinical studies can further explore the treatment of PPD through the combined use of microorganisms. This not only offers insights into the pathogenesis of PPD but also lays the foundation for utilizing gut microbiota as biotherapeutics in treating neurological disorders.

## 1 Introduction

Postpartum depression (PPD) represents a grave mental health concern, typically manifesting within a week to several weeks following childbirth. Beyond the evident physical and psychological toll on the mother, PPD has far-reaching implications for the child, potentially hampering their emotional, behavioral, cognitive, and intellectual trajectory in ways that can span their lifetime (Josefsson et al., 2002; Sohr-Preston and Scaramella, 2006; van der Waerden et al., 2017). Globally, it’s estimated that 10–15% of new mothers grapple with PPD, although the prevalence fluctuates across regions (Palumbo et al., 2017). Alarmingly, the prevalence is heightened in developing nations. Countries with the lowest incidence rates of PPD include: Singapore (3%), the Netherlands (8%), the United States (8.4%), and Switzerland (11%). Countries with higher incidence rates include: Chile (38%), South Africa (37%), Turkey (28%), and China (21.4%) (Hahn-Holbrook et al., 2017; Liu et al., 2022).

The human gut is a bustling microcosm, In the case of an average adult weighing 70 kg, the gut microbiota comprises approximately 3.8 × 10^13^ microorganisms, collectively possessing genomic content that dwarfs our own by over 100-fold (Gill et al., 2006; Sender et al., 2016). The human gut microbiota is composed of four major phyla: Firmicutes, Bacteroidetes, Proteobacteria, and Actinobacteria (Eckburg et al., 2005). In some cohort studies, compared to healthy controls (HC), patients with Major Depressive Disorder (MDD) exhibit higher abundances of Bacteroidetes, Proteobacteria, and Actinobacteria (Jiang et al., 2015). In patients with PPD, a relatively lower abundance of the Firmicutes phylum is observed (Zhou et al., 2020). A burgeoning body of evidence points to intricate bidirectional communication between our brains and gut, with the microbial denizens and their metabolic by-products playing pivotal roles. This intricate dialogue is called the brain-gut-microbiota axis (Mayer et al., 2014; Cryan et al., 2019). In patients with depression, the homeostasis of the gut microbiota is disrupted, leading to impaired gut function. This, in turn, results in intestinal barrier dysfunction and various inflammatory responses (Liu et al., 2023). These inflammatory responses are correlated with the pathogenesis of depression. The brain-gut axis is bidirectional, with the vagus nerve being the primary regulatory pathway between the brain and the gut microbiota. Some studies suggest that subdiaphragmatic vagotomy may reduce inflammatory responses, thereby alleviating depressive symptoms (Zhang et al., 2020; Pu et al., 2021). Clinically, vagal nerve stimulation (VNS) is an FDA-approved neuromodulation therapy for the treatment of severe treatment-resistant depression (TRD). VNS increases the levels of serotonin (5-hydroxytryptamine, 5-HT) and norepinephrine, and through the anti-inflammatory pathway of the vagus nerve, it reduces systemic inflammatory responses, thereby improving depressive symptoms (Kamel et al., 2022). Short-chain fatty acids (SCFAs) act as intermediary products between the gut microbiota and the brain, primarily including acetate, propionate, and butyrate. SCFA produced by gut microbiota inhibit histone deacetylases and activate G-protein-coupled receptors, influencing systemic physiological responses (Tan et al., 2014). Among these, propionate can reduce levels of γ-aminobutyric acid (GABA), and the indoleamine serotonin (El-Ansary et al., 2012). During pregnancy, alterations in the GABA(A) receptor may increase neuronal excitability in the brain. However, the sharp decrease in neuroactive steroids in the brain after childbirth has a causal relationship with the expression of GABA(A) receptors in the hippocampus. This dynamic could contribute to the development of postpartum neurological and psychiatric disorders (Maguire et al., 2009). Regulating the gut microbiota can improve intestinal barrier function, reduce systemic inflammatory responses, and increase the abundance of beneficial bacteria in the gut. This enhances their ability to produce beneficial metabolites such as SCFAs and GABA, which have positive effects on alleviating and treating postpartum depression (Ramsteijn et al., 2020; Tian et al., 2021).

Mendelian Randomization (MR), rooted in epidemiology, leverages single nucleotide polymorphisms (SNPs) identified in genome-wide association studies (GWAS) as instrumental variables (IVs) to estimate the causal relationship between exposure factors and disease outcomes. These SNPs are genetically associated with the exposure factors but are not influenced by confounding factors and reverse causation. The advantage of MR is that these instrumental variables are predetermined conceptually, greatly minimizing the impact of potential confounders and reverse causality (Ellervik et al., 2019). Recent studies have applied MR to analyze the causal relationship between psychiatric disorders and the gut microbiome. Actinobacteria, *Bifidobacterium*, *Ruminococcus1*, and *Streptococcaceae* have been associated with MDD (Chen et al., 2022), *Prevotellaceae* with autism spectrum disorder, Betaproteobacteria with bipolar disorder (Ni et al., 2021). Despite the presence of symptomatic and genetic parallels between PPD and MDD, substantial disparities in their etiology and treatment methodologies are evident (Jairaj and Rucker, 2022).

Therefore, this study aims to use the MR method to analyze the potential causal relationships between various levels of gut microbiota and PPD, to explore the differences in the gut microbiota between PPD and MDD, and other psychiatric disorders, thus providing new insights into treatment approaches for PPD.

## 2 Materials and methods

### 2.1 Mendelian randomization analysis

MR provides one method for assessing the causal nature of some exposures (Smith and Ebrahim, 2003; Lawlor et al., 2008). In epidemiological research, MR harnesses genetic variants as instrumental variables (IVs) to decipher the causal interplay between exposure and its consequent outcome (Bowden and Holmes, 2019).

To perform an MR study, three main assumptions must be fulfilled (Konig and Greco, 2018). (1) There must be a strong correlation between the genetic variant and the exposure; (2) the genetic instrument is independent of potential confounders of the exposure-outcome association; (3) the genetic variant should be solely associated with the outcome through the exposure, without being influenced by other confounders (Haycock et al., 2016; Konig and Greco, 2018; Hirtz et al., 2022).

### 2.2 Study design

**Figure 1:**
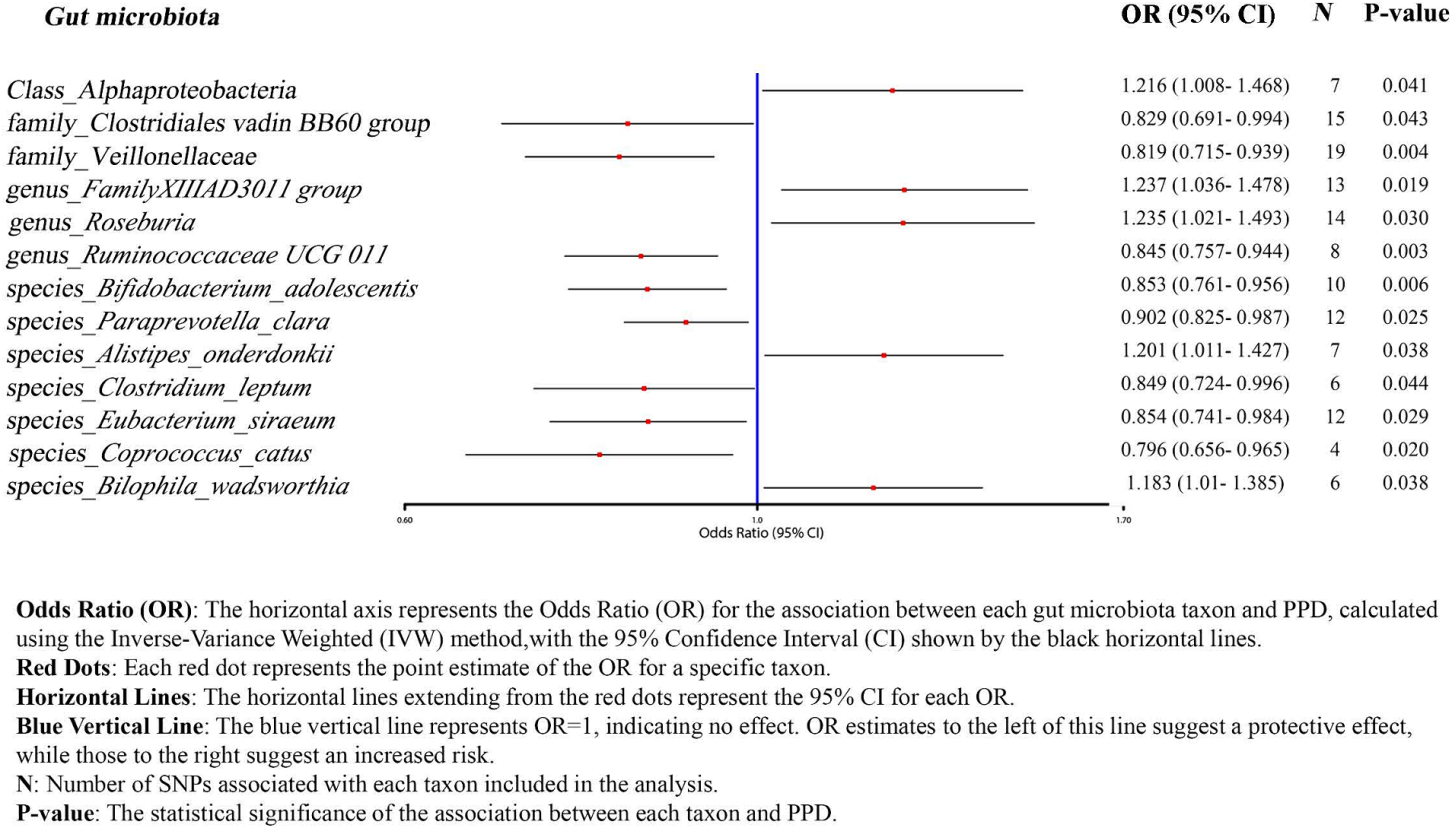
Overview of the analysis process of the causal relationship between the gut microbiota and PPD through MR analysis.

In this research design, gut microbiota SNPs are taken as the exposure variable, and PPD is considered as the outcome variable. Figure 1 illustrates the detailed flowchart.

### 2.3 Data source

Summary-level GWAS data related to the human gut microbiota from the international consortium MiBioGen (Kurilshikov et al., 2021) and the Dutch Microbiome Project (DMP) (Lopera-Maya et al., 2022). GWAS summary-level data on PPD were obtained from the FinnGen biobank, comprising a total of 67,205 individuals of European ancestry, including 7,604 cases, 59,601 controls. The detailed descriptions of exposure and outcome, including the data source are presented in Table 1.

**Table 1:** Information of the data source for gut microbiota and PPD.

Variables	Consortium	Traits	Sample size	nSNPs	nTaxa	Websites
Exposure	MiBioGen	Phylum	18,340		9	https://mibiogen.gcc.rug.nl/menu/main/home
		Class			16	
		Order			20	
		Family			35	
		Genus			131	
	DMP	Species	8,208		105	https://dutchmicrobiomeproject.molgeniscloud.org/menu/main/home
Outcome	FinnGen	PPD	67,205	16,376,275		https://www.finngen.fi/en

### 2.4 Selection of instrumental variable

To ensure the accuracy and reliability of the causal effect between gut microbiota and PPD, we adopt the following quality control methods to select microbiota-related IVs:

1. Based on previous experience and to obtain more comprehensive results, we select IVs with a significance threshold of *p* < 10^−5^.
2. To rule out the influence of linkage disequilibrium on the results, we used PLINK software with threshold values set at clump_kb = 5,000 and clump_r^2^ = 0.001.
3. To ensure that SNPs for exposure and outcome are influenced by the same alleles, we are excluding palindromic SNPs from IVs.
4. The first assumption of MR can be directly validated by calculating the *F*-statistic. An *F*-statistic greater than 10 indicates the absence of weak instrument bias (*F* for a single SNP equals *β*^2^/SE^2^) (Hirtz et al., 2022).

### 2.5 Mendelian randomization methods

In the current study, all analyses were performed using R software version 4.3.2, utilizing the “Mendelian-Randomization” package. Five popular MR methods were used: Inverse-variance weighted (IVW), MR-Egger, weighted median estimator, simple modal-based estimation, and the weighted mode method. Each statistical method operates under its own set of methodological assumptions. The IVW method estimates the overall causal effect of an exposure on an outcome by performing a weighted average of the effects of multiple genetic variants. This approach assumes the absence of horizontal pleiotropy (Burgess et al., 2013). The MR-Egger assumes the presence of pleiotropy in >50% of SNPs (Bowden et al., 2015). When less than 50% of the information comes from invalid IVs, the weighted median estimator offers a consistent estimate of causal effects (Bowden et al., 2016). While the modal-based estimate (MBE) has a weaker ability to detect causal effects, it relaxes instrumental variable assumptions and requires a smaller sample size (Hartwig et al., 2017). Under different methodological assumptions, IVW results are more reliable in the absence of heterogeneity and pleiotropy. In the presence of heterogeneity but no pleiotropy, the WM method is more reliable. When pleiotropy exists, the MR-Egger method performs better (Burgess and Thompson, 2017).

To satisfy the third assumption of MR and ensure the accuracy and stability of the results, further sensitivity analysis was conducted:

1. Cochran’s *Q* test is used to quantify the heterogeneity between SNPs. A *p*-value less than 0.05 indicates significant heterogeneity (Bowden et al., 2019).
2. MR-PRESSO and MR-Egger regression tests are employed to detect potential horizontal pleiotropic effects. If the intercept term is significant, it means the existence of horizontal multiplicity. Compared to MR-Egger, MR-PRESSO offers higher accuracy (Verbanck et al., 2018).
3. The leave-one-out sensitivity analysis is adopted to assess the stability of the results.

## 3 Results

### 3.1 Instrumental variables selection

Detailed information for 3,509 IVs is presented in Supplementary Table S1 (*p*-value < 1e−5). These IVs are categorized into 9 phyla (121 SNPs, range 10–18), 20 orders (275 SNPs, range 5–19), 16 classes (219 SNPs, range 8–22), 33 families (438 SNPs, range 5–21), 119 genera (1,511 SNPs, range 3–26), 105 species (945 SNPs, range 1–17). The *F*-statistic of each IV was greater than 10 (Supplementary Table S1).

### 3.2 Two-sample MR

**Figure 2:**
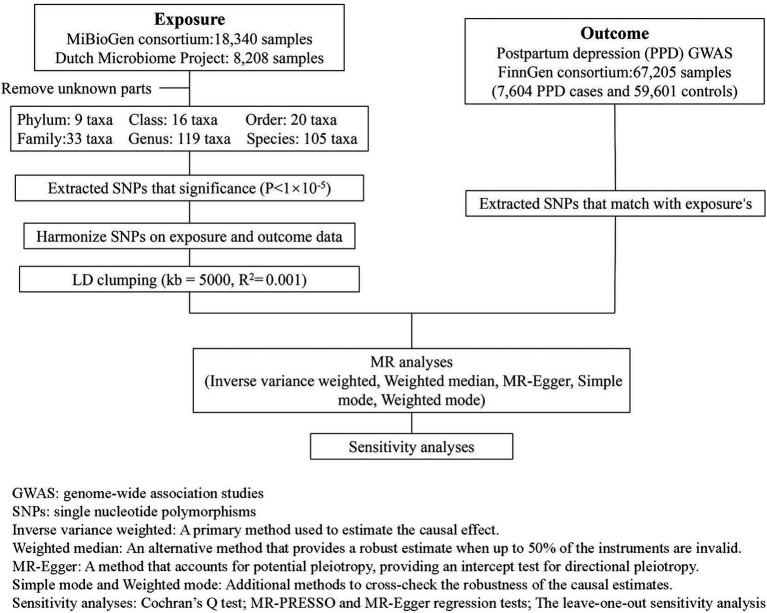
Forrest plot results from MR between the gut microbiota and PPD risk.

We employed IVW as the primary analysis method for MR. The results indicate an association between the risk of PPD and 13 genetically predicted bacterial taxa (Figure 2). The results are presented in Table 2.

**Table 2:** Full result of MR estimates for the association between gut microbiota and PPD.

Level	Bacterial taxa	Method	nSNP	Bate	SE	OR (95% CI)	*p*-value
Class	Alphaproteobacteria	MR Egger	7	0.595	0.358	1.812 (0.898–3.656)	0.158
		Weighted median		0.177	0.121	1.194 (0.943–1.513)	0.142
		Inverse variance weighted		0.196	0.096	1.216 (1.008–1.468)	0.041
		Simple mode		0.165	0.178	1.179 (0.832–1.671)	0.390
		Weighted mode		0.181	0.170	1.198 (0.86–1.671)	0.327
family	Clostridiales vadin BB60 group	MR Egger	15	−0.010	0.257	0.99 (0.598–1.639)	0.970
		Weighted median		−0.200	0.099	0.819 (0.675–0.994)	0.043
		Inverse variance weighted		−0.188	0.093	0.829 (0.691–0.994)	0.043
		Simple mode		−0.226	0.141	0.798 (0.606–1.051)	0.131
		Weighted mode		−0.208	0.121	0.813 (0.641–1.03)	0.108
family	Veillonellaceae	MR Egger	19	−0.258	0.141	0.773 (0.586–1.019)	0.085
		Weighted median		−0.234	0.105	0.791 (0.644–0.971)	0.025
		Inverse variance weighted		−0.199	0.070	0.819 (0.715–0.939)	0.004
		Simple mode		−0.334	0.173	0.716 (0.51–1.005)	0.069
		Weighted mode		−0.270	0.123	0.764 (0.6–0.973)	0.042
genus	*FamilyXIIIAD3011 group*	MR Egger	13	−0.078	0.427	0.925 (0.401–2.134)	0.859
		Weighted median		0.234	0.120	1.263 (0.999–1.598)	0.051
		Inverse variance weighted		0.213	0.091	1.237 (1.036–1.478)	0.019
		Simple mode		0.309	0.208	1.362 (0.906–2.048)	0.163
		Weighted mode		0.301	0.184	1.352 (0.943–1.938)	0.127
genus	*Roseburia*	MR Egger	14	0.472	0.297	1.604 (0.896–2.872)	0.138
		Weighted median		0.234	0.138	1.264 (0.965–1.655)	0.088
		Inverse variance weighted		0.211	0.097	1.235 (1.021–1.493)	0.030
		Simple mode		0.207	0.243	1.23 (0.764–1.981)	0.410
		Weighted mode		0.240	0.223	1.271 (0.82–1.969)	0.302
genus	*Ruminococcaceae UCG 011*	MR Egger	8	0.230	0.279	1.258 (0.728–2.175)	0.442
		Weighted median		−0.158	0.078	0.854 (0.733–0.994)	0.042
		Inverse variance weighted		−0.168	0.056	0.845 (0.757–0.944)	0.003
		Simple mode		−0.152	0.111	0.859 (0.692–1.068)	0.213
		Weighted mode		−0.145	0.111	0.865 (0.696–1.074)	0.231
species	*Bifidobacterium adolescentis*	MR Egger	10	−0.109	0.220	0.896 (0.583–1.379)	0.632
		Weighted median		−0.144	0.080	0.866 (0.741–1.012)	0.070
		Inverse variance weighted		−0.159	0.058	0.853 (0.761–0.956)	0.006
		Simple mode		−0.086	0.130	0.917 (0.711–1.184)	0.524
		Weighted mode		−0.112	0.113	0.894 (0.716–1.115)	0.346
Species	*Paraprevotella clara*	MR Egger	12	0.154	0.186	1.166 (0.81–1.679)	0.428
		Weighted median		−0.102	0.062	0.903 (0.8–1.02)	0.099
		Inverse variance weighted		−0.103	0.046	0.902 (0.825–0.987)	0.025
		Simple mode		−0.205	0.122	0.815 (0.641–1.035)	0.121
		Weighted mode		−0.203	0.116	0.816 (0.65–1.025)	0.108
Species	*Alistipes onderdonkii*	MR Egger	7	−0.085	0.451	0.919 (0.379–2.225)	0.859
		Weighted median		0.093	0.119	1.097 (0.87–1.385)	0.434
		Inverse variance weighted		0.183	0.088	1.201 (1.011–1.427)	0.038
		Simple mode		0.038	0.188	1.039 (0.719–1.501)	0.845
		Weighted mode		0.036	0.193	1.037 (0.71–1.515)	0.858
Species	*Clostridium leptum*	MR Egger	6	0.191	0.438	1.211 (0.513–2.857)	0.685
		Weighted median		−0.107	0.079	0.899 (0.77–1.049)	0.177
		Inverse variance weighted		−0.163	0.081	0.849 (0.724–0.996)	0.044
		Simple mode		−0.094	0.111	0.91 (0.732–1.131)	0.435
		Weighted mode		−0.064	0.109	0.938 (0.757–1.161)	0.580
Species	*Eubacterium siraeum*	MR Egger	12	−0.421	0.328	0.656 (0.345–1.249)	0.229
		Weighted median		−0.101	0.090	0.904 (0.757–1.079)	0.265
		Inverse variance weighted		−0.158	0.072	0.854 (0.741–0.984)	0.029
		Simple mode		−0.053	0.168	0.948 (0.683–1.317)	0.757
		Weighted mode		−0.050	0.147	0.951 (0.713–1.27)	0.741
Species	*Coprococcus catus*	MR Egger	4	−0.344	0.388	0.709 (0.331–1.516)	0.469
		Weighted median		−0.256	0.126	0.774 (0.605–0.99)	0.041
		Inverse variance weighted		−0.229	0.099	0.796 (0.656–0.965)	0.020
		Simple mode		−0.271	0.168	0.763 (0.548–1.061)	0.206
		Weighted mode		−0.289	0.150	0.749 (0.558–1.005)	0.149
Species	*Bilophila wadsworthia*	MR Egger	6	0.116	0.226	1.123 (0.721–1.748)	0.636
		Weighted median		0.163	0.100	1.177 (0.967–1.432)	0.104
		Inverse variance weighted		0.168	0.081	1.183 (1.01–1.385)	0.038
		Simple mode		0.157	0.130	1.17 (0.908–1.508)	0.280
		Weighted mode		0.161	0.133	1.175 (0.905–1.525)	0.280

Using the IVW method as the primary analysis, the results indicated that 13 bacterial taxa were associated with the risk of PPD when the significance level was less than 0.05. Specifically, the following taxa were inversely associated with the risk of PPD: Clostridiales vadin BB60 group, Veillonellaceae (at family level); *Ruminococcaceae UCG 011* (at genus level); *Bifidobacterium adolescentis*, *Paraprevotella clara*, *Clostridium leptum*, *Eubacterium siraeum*, *Coprococcus catus* (at species level). Whereas the following taxa may be associated with a higher risk of PPD: Alphaproteobacteria (at class level); *FamilyXIIIAD3011 group, Roseburia* (at genus level); *Alistipes onderdonkii*, *Bilophila wadsworthia* (at species level) (Figure 2; Table 2). The scatter plots (Supplementary Figure S1) and forest plots (Supplementary Figure S2) of the above results demonstrate the stability of the findings.

Table 3 shows that the instrumental variable of Clostridiales vadin BB60 group has a significant heterogeneity with the outcome by Cochran’s *Q* test (*p* < 0.05), and the others were no significant heterogeneity identified among the SNPs (*p* > 0.05).

**Table 3:** The heterogeneity results from the Cochran’s *Q* test.

No	Level	Bacterial taxa	MR-Egger		IVW	
			*Q*	*p* value	*Q*	*p* value
1	Class	Alphaproteobacteria	0.866	0.973	2.202	0.900
2	Family	Clostridiales vadin BB60 group	24.069	0.031	25.092	0.034
3	Family	Veillonellaceae	14.444	0.635	14.673	0.684
4	Genus	*FamilyXIIIAD3011 group*	7.184	0.784	7.671	0.810
5	Genus	*Roseburia*	11.678	0.472	12.547	0.483
6	Genus	*Ruminococcaceae UCG 011*	4.993	0.545	7.110	0.418
7	Species	*Bifidobacterium adolescentis*	6.022	0.645	6.077	0.732
8	Species	*Paraprevotella clara*	9.865	0.452	11.879	0.373
9	Species	*Alistipes onderdonkii*	3.482	0.626	3.848	0.697
10	Species	*Clostridium leptum*	9.267	0.055	10.847	0.055
11	Species	*Eubacterium siraeum*	13.926	0.176	14.865	0.189
12	Species	*Coprococcus catus*	0.803	0.669	0.898	0.826
13	Species	*Bilophila wadsworthia*	1.168	0.883	1.229	0.942

Table 4 shows that there was no demonstrated pleiotropy detected in the MR Egger test (*p* < 0.05). MR-PRESSO suggests that Clostridiales vadin BB60 group has horizontal pleiotropy with outcome (*p* = 0.04, Table 3). According to the findings of the leave-one-out analysis, no SNPs were found to have affected causal association (Supplementary Figure S3).

**Table 4:** Pleiotropy results from Egger intercept and PRESSO analysis.

No	Level	Bacterial taxa	Egger *p* value	PRESSO *p* value
1	Class	Alphaproteobacteria	0.300	0.91
2	Family	Clostridiales vadin BB60 group	0.470	0.04
3	Family	Veillonellaceae	0.638	0.743
4	Genus	*FamilyXIIIAD3011 group*	0.500	0.855
5	Genus	*Roseburia*	0.370	0.502
6	Genus	*Ruminococcaceae UCG 011*	0.196	0.501
7	Species	*Bifidobacterium adolescentis*	0.820	0.756
8	Species	*Paraprevotella clara*	0.186	0.414
9	Species	*Alistipes onderdonkii*	0.572	0.700
10	Species	*Clostridium leptum*	0.455	0.098
11	Species	*Eubacterium siraeum*	0.431	0.228
12	Species	*Coprococcus catus*	0.788	0.803
13	Species	*Bilophila wadsworthia*	0.818	0.956

## 4 Discussion

Our MR analysis further illuminated a nominal causal bond linking PPD with 13 distinct microbial taxa. To ensure the transparency and completeness of our Mendelian Randomization study report, we adopted the STROBE-MR guidelines (Supplementary Table S2). STROBE-MR is a set of reporting guidelines specifically designed for Mendelian Randomization studies, consisting of 20 items. We have provided the complete STROBE-MR checklist in the appendix for readers’ reference.

Observational research findings indicate a decrease in the abundance of the *Faecalibacterium* genus in patients with PPD (Zhou et al., 2020), a phenomenon that aligns with the negative correlation we observed with *Ruminococcaceae UCG 011* through our MR analysis. *Ruminococcaceae* portray a noticeable reduction in individuals with pronounced depression (Radjabzadeh et al., 2022). In the realm of animal studies, they exhibit a positive association with vital sugar metabolic cascades, such as gluconeogenesis, glycolysis, and the pentose phosphate trajectory (Zhang et al., 2019). Given that obesity is one of the comorbidities of depression, we hypothesize that it might indirectly affect PPD. Within *Ruminococcaceae*, *Faecalibacterium prausnitzii* is one of the main bacteria in the human gut and converts acetate to butyrate through the butyryl-CoA: acetate CoA-transferase (Duncan et al., 2002). This mechanism could potentially account for the diminished abundance of the *Faecalibacterium* genus observed in patients with PPD.

Previous MR studies on the gut microbiota and MDD indicated that the Actinobacteria class, *Bifidobacterium* genus, and *Ruminococcus1* genus had a protective effect against MDD (Chen et al., 2022). This aligns with our findings for *Ruminococcaceae UCG 011* (OR 0.845, 95%CI 0.757–0.944) and *Bifidobacterium adolescentis* (OR 0.853, 95%CI 0.761–0.956). *Bifidobacterium*, the most common microbe within Actinobacteria in the human gut, is also a widely-used probiotic. A study demonstrated that *Bifidobacterium* can alleviate depression and gut-related diseases by altering the gut microbiota and its tryptophan metabolism (Tian et al., 2022). Postpartum, women experience an increase in the abundance of *Bifidobacterium*, a change that may also enhance the production of short-chain fatty acids, thereby aiding in the establishment and refinement of the neonatal gut microbiota environment (Qin et al., 2022). Given the symptomatic similarities between PPD and MDD, *Bifidobacterium* may also have the potential to treat or prevent PPD.

Research has shown that good mental health in mothers can reduce anxiety and mitigate stress responses, which is crucial for the successful delivery and upbringing of offspring (Hillerer et al., 2011). The hippocampus, a limbic structure involved in emotion and cognition, plays a critical role in these processes. Within the hippocampus, brain-derived neurotrophic factor (BDNF) and its receptor, TrkB (tyrosine kinase receptor B), are directly involved in various physiological functions of the central nervous system, such as neuronal survival, synaptic plasticity, and learning and memory (Leibrock et al., 1989; von Bohlen und Halbach, 2010). During pregnancy, if a mother is subjected to chronic stress or chronic restraint stress (CRS), the expression of BDNF and TrkB in the hippocampus can be significantly reduced. This reduction is believed to be one of the contributing factors to the high incidence of depression during pregnancy and postpartum (Ye et al., 2011). Notably, repeated restraint stress in the final week of pregnancy can induce depressive-like behaviors in mothers (O'Mahony et al., 2006).

The administration of *Bifidobacterium adolescentis* in mice subjected to CRS not only reduces anxiety and depressive-like behaviors and increases BDNF expression levels, but also reverses the dysbiosis of the gut microbiota induced by CRS (Guo et al., 2019). Additionally, *Bifidobacterium longum* has been shown to significantly reduce depressive-like behaviors in the forced swim test (FST) and decrease anxiety-like behaviors in the open field test (OFT) (Savignac et al., 2014). The combined use of *Bifidobacterium longum* and *Lactobacillus helveticus* has been found to alleviate symptoms of anxiety and depression in healthy individuals (Radford-Smith and Anthony, 2023).

Within the MR assessment, particular bacterial groups, Veillonellaceae (OR 0.819, 95%CI 0.715–0.939), *Paraprevotella clara* (OR 0.902, 95%CI 0.825–0.987), *Clostridium leptum* (OR 0.849, 95%CI 0.724–0.996), *Eubacterium siraeum* (OR 0.854, 95%CI 0.741–0.984), *Coprococcus catus* (OR 0.796, 95%CI 0.656–0.965), emerged as potentially protective entities.

Veillonellaceae, as non-fermentative microbes, distinguish themselves with their propensity to modulate the enzymatic activity of methylmalonyl-CoA decarboxylase, thereby playing a pivotal role in fatty acid metabolism. Noteworthy is their marked depletion in autism-afflicted individuals, potentially influencing lactate fermentation and specific short-chain fatty acid synthesis (Gronow et al., 2010; Wang et al., 2023). They also show a negative correlation with psychological stress (Ma et al., 2023).

*Paraprevotella clara* can reduce the levels of trypsin in the colon, thereby decreasing the likelihood of developing inflammatory bowel diseases, while also offering protection against certain viral infections in the colon (Li et al., 2022). Intestinal inflammation may reduce gut permeability, leading to the translocation of microbes originally present in the intestinal tract into the peripheral circulation, thereby disrupting normal brain function and affecting specific behaviors (D'Mello et al., 2015). This is consistent with our research findings that *Paraprevotella clara* exhibits a negative correlation with PPD.

Due to a lack of experimental studies, the mechanisms by which *Clostridium leptum*, *Coprococcus catus*, and *Eubacterium siraeum* affect PPD remain unclear. In a survey study of 1,070 patients with depression, *Coprococcus* was found to be nearly depleted in individuals with depression. *Coprococcus* possesses biosynthetic pathways related to dopamine synthesis and participates in the synthesis of GABA, improving the quality of psychological life (Valles-Colomer et al., 2019). *Clostridium leptum* is associated with glucose metabolism (Palmnas-Bedard et al., 2022), leading us to speculate that *Clostridium leptum* may be related to GABA produced during the body’s glucose metabolic processes. However, this hypothesis has not yet been validated.

Five positive results were identified: Alphaproteobacteria (OR 1.216, 95%CI 1.008–1.468), *Roseburia* (OR 1.235, 95%CI 1.021–1.493), *FamilyXIIIAD3011group* (OR 1.237, 95%CI 1.036–1.478), *Alistipes onderdonkii* (OR 1.201, 95%CI 1.011–1.427), *Bilophila wadsworthia* (OR 1.183, 95%CI 1.01–1.385). A surge in Alphaproteobacteria’s presence typically heralds a perturbation in the gut’s ecological equilibrium (Litvak et al., 2017). Previous research insights spotlight Rhodospirillaceae, a member of the Alphaproteobacteria phylum, underscoring its positive correlation with the genesis of Alzheimer’s disease (Zhou et al., 2021). Therefore, we infer that an increase in the abundance of Alphaproteobacteria may signal changes in the gut ecological environment, thereby affecting the normal growth of other bacterial communities and the composition of their metabolic products.

*Roseburia* stands as a significant regulator in the realms of gut microbiota ecology, immune modulation, and neurological afflictions. Within this genus, five species notably dominate: *Roseburia intestinalis*, *R. hominis*, *R. inulinivorans*, *R. faecis*, and *R. cecicola* (Tamanai-Shacoori et al., 2017). Of these, *R. intestinalis* holds a particular distinction, constituting between 0.9 and 5.0% of the entire microbial community and acting as the chief butyrate producer (Hold et al., 2003). In an insightful study, colitis-afflicted rats treated with *R. intestinalis* demonstrated a diminished display of anxiety and depressive-like symptoms. The underlying mechanism could be *R. intestinalis*’ influence on 5-hydroxytryptamine (5-HT) expression in colonic regions. As a neurotransmitter, 5-HT plays a pivotal role in mediating the gut-brain axis, influencing brain functionality (Xu et al., 2021). While the abundance of *R. intestinalis* is lower in patients with depression compared to the healthy population (Zheng et al., 2016), other studies have shown a higher abundance of *R. intestinalis* in patients with MDD compared to healthy individuals (Jiang et al., 2015). These findings highlight that while *Roseburia* does play a role in neurological diseases, its interaction mechanisms are intricate. Furthermore, Reserpine can inhibit the central nervous system’s regular functions by altering catecholamines and 5-HT levels in brain tissues, leading to depressive behaviors (Yan et al., 2023). Taking into account *Roseburia*’s position as a positive determinant in our MR analysis, it’s plausible to hypothesize that an overabundance of *Roseburia* might interfere with levels of serotonergic depression markers like 5-HT, thereby affecting the functionality of the nervous system.

An increase in the abundance of *Bilophila wadsworthia* can disrupt hippocampal synaptic plasticity, neurogenesis, and gene expression. Ketogenic diets and hypoxic environments can alter the abundance of *Bilophila wadsworthia*, thereby impairing cognitive behavior (Olson et al., 2021). However, some studies have found that ketogenic diets can increase GABA levels. Currently, there is no definitive clinical data to indicate whether ketogenic diets can improve or exacerbate PPD (Wlodarczyk et al., 2021).

*Alistipes onderdonkii* is a relatively new bacterium identified to increase in abundance under conditions of high stress or prolonged fatigue. PPD often involves extended periods of battling fatigue or stress. The increase in *Alistipes* decreases serotonin availability and disrupts the gut-brain axis (Parker et al., 2020).

Previous research has conducted numerous analyses on the association between the gut microbiome and psychiatric disorders. Although PPD shares certain symptomatic and genetic similarities with other mental health conditions, the pathogenesis of PPD is influenced by multiple factors, particularly fluctuations in hormone levels. Therefore, our MR analysis has the potential to reveal more causal relationships, paving the way for future research into bacterial biosensor detection and therapeutic interventions for related diseases.

This study possesses several strengths and limitations. Employing the MR method to analyze the causal relationship between PPD and the gut microbiota minimizes interference from confounding factors. Techniques such as MR-PRESSO and MR-Egger were utilized to eliminate the effects of horizontal pleiotropy and heterogeneity, ensuring the accuracy of the results. This study refined the classification of the gut microbiome down to the species level, making the results more comprehensive and detailed compared to previous research. However, the study also has its limitations; the GWAS data derived from the MiBioGen consortium, DMP, and FinnGen consortium, may introduce selection bias. Moreover, the data primarily originates from European populations, hence extrapolating these results to diverse populations should be approached with caution. The etiological factors behind PPD remain enigmatic, alluding to the existence of potential undisclosed confounders. Despite employing various methods for sensitivity analysis, the impact of horizontal pleiotropy could not be fully assessed. Lastly, *in vitro* experimental validation was not conducted. In future studies, we will strengthen the functional validation through related experiments to further substantiate our research findings.

In conclusion, with the continuous development of medical statistical techniques and the refinement of GWAS data, future studies should focus on the integrated development of multiple disciplines and omics, exploring complex diseases, genetic variations, and environmental changes at various levels of interaction.

## Data Availability

The original contributions presented in the study are included in the article/Supplementary material, further inquiries can be directed to the corresponding author.
