# Supplementary material for: The causal association between gut microbiota and postpartum depression: a two-sample Mendelian randomization study
Source: Front Microbiol. 2024 Sep 2;15:1415237. doi: 10.3389/fmicb.2024.1415237 (PMC11402819; doi:10.3389/fmicb.2024.1415237)
Supplement: Supplementary file 3 [file Data_Sheet_1.DOCX]

Supplementary Material

# Supplementary Figures

**
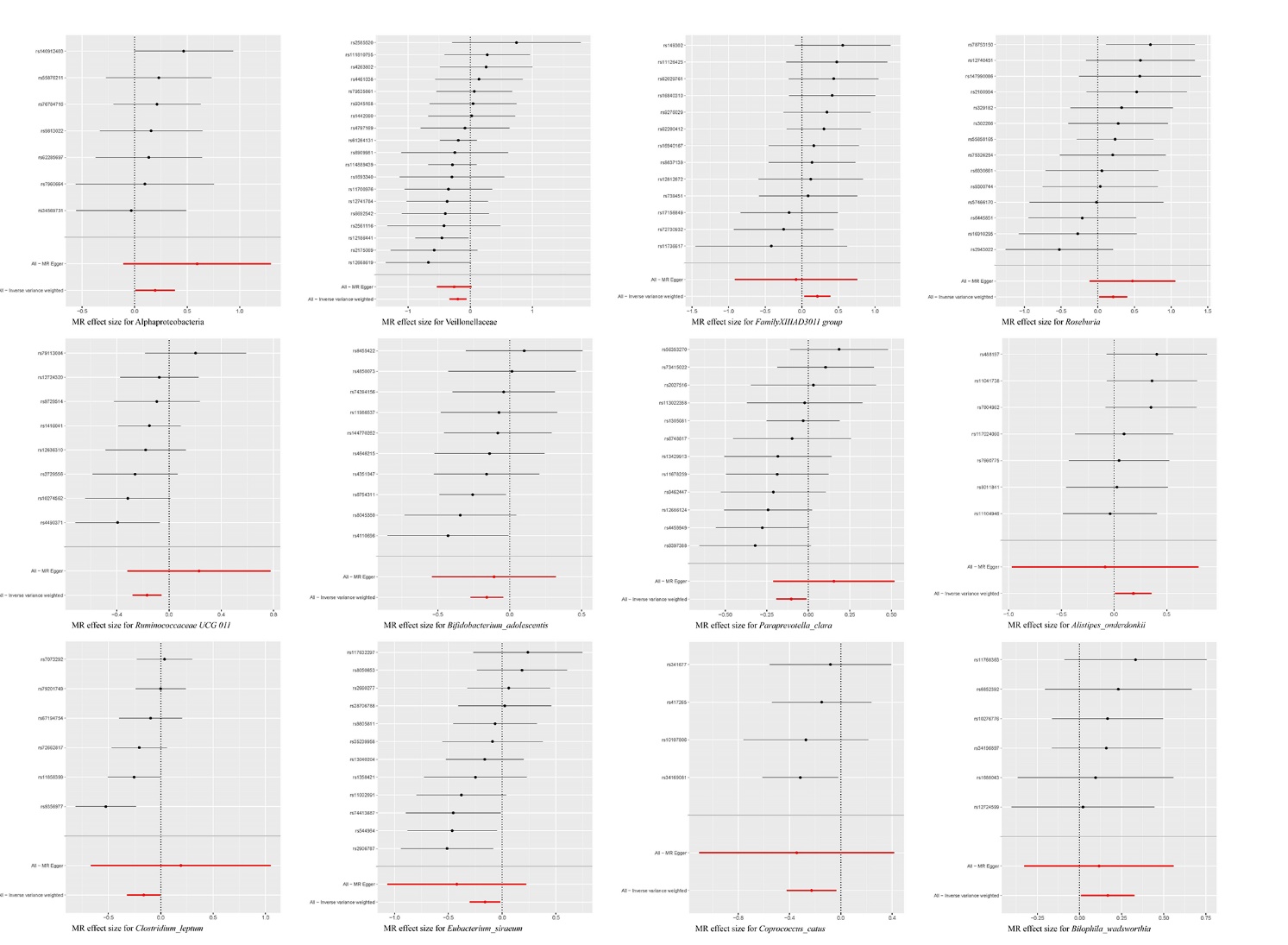
**

**Supplementary Figure 1.** The forest plots for the association between gut microbiota and PPD.


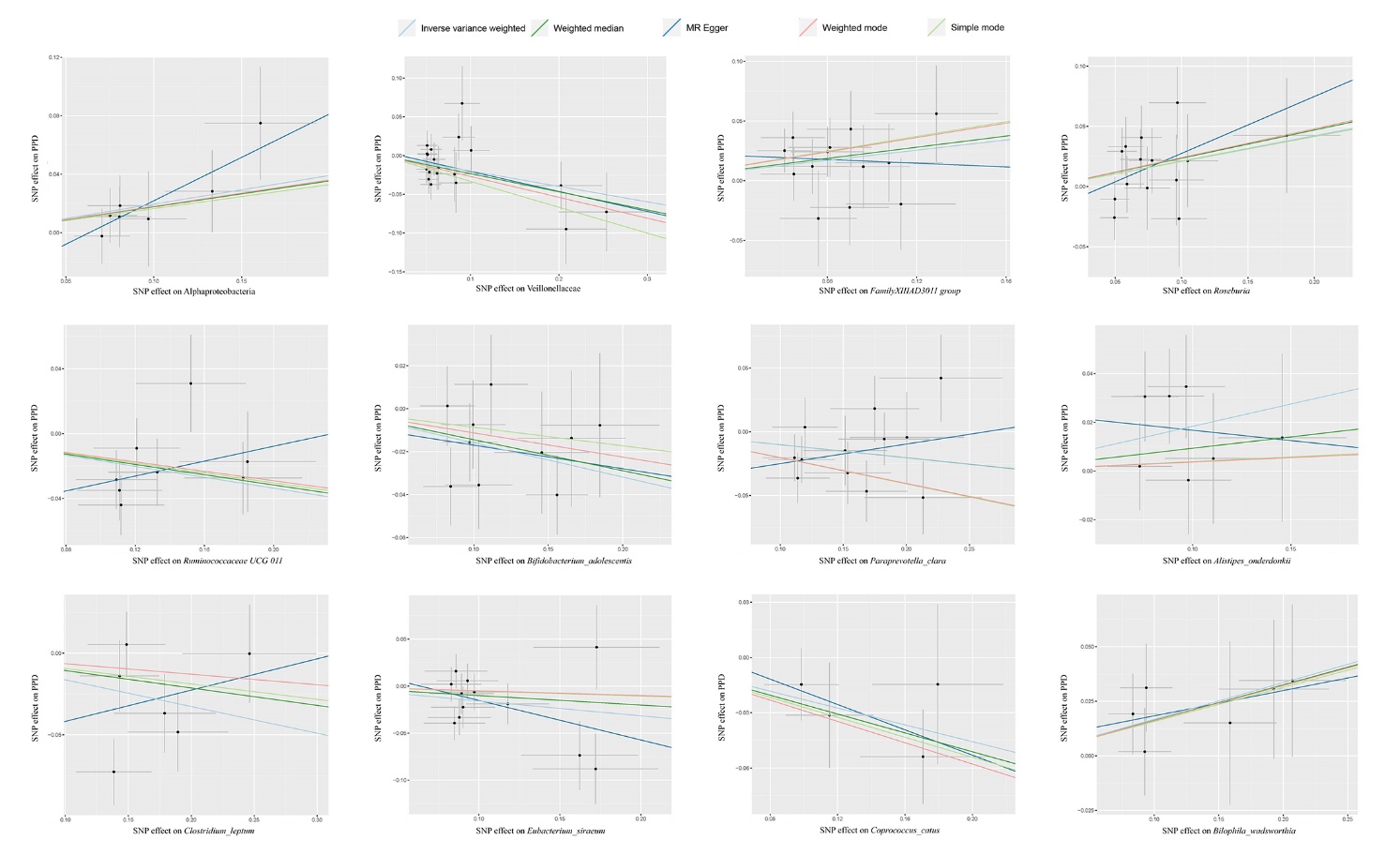
 **Supplementary Figure 2.** The scatter plots for association between gut microbiota and PPD.


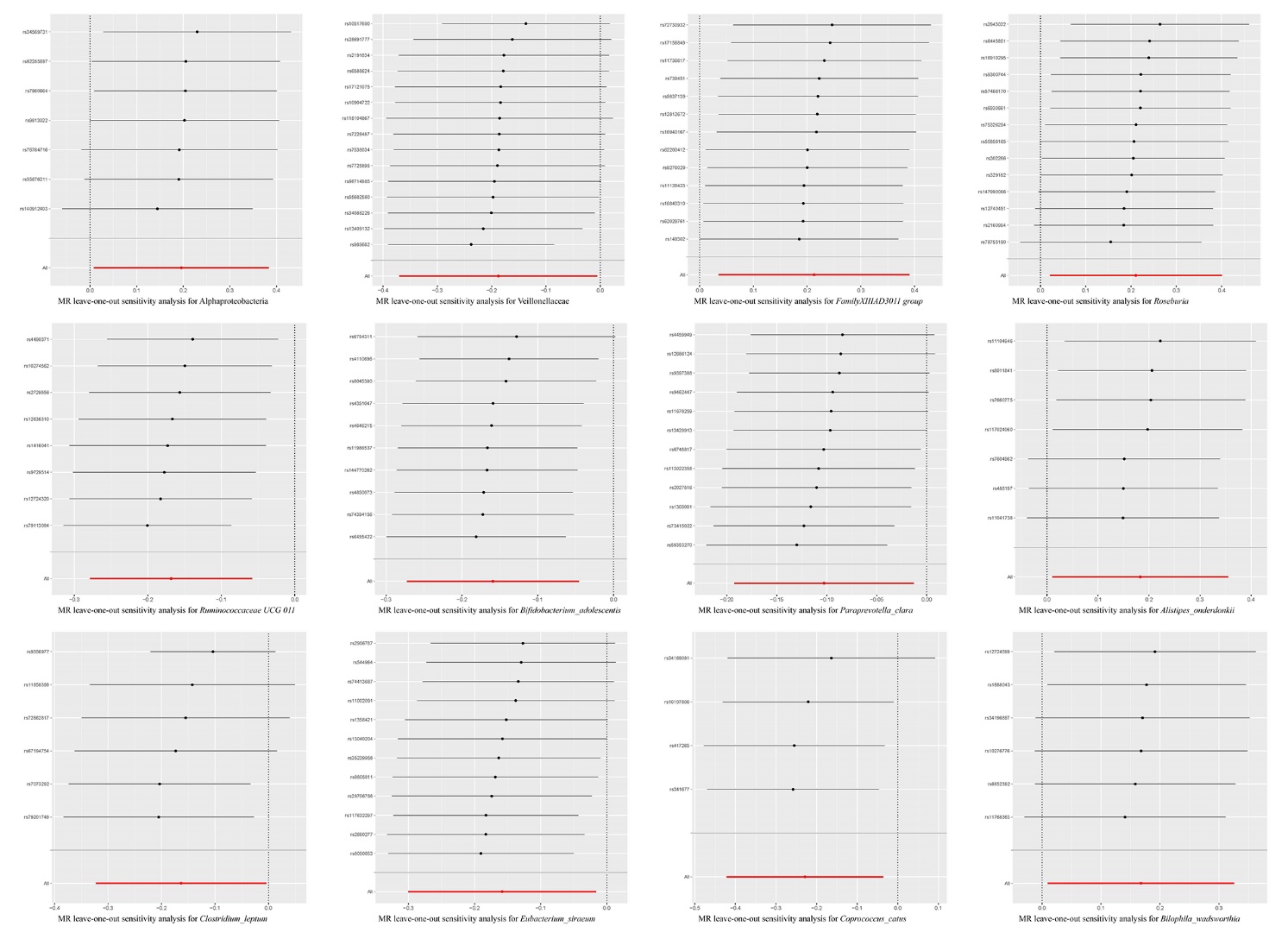
 **Supplementary Figure 3.** The leave-one-out sensitivity analysis for the association between gut microbiota and PPD.
